# Trends and causes of maternal mortality in Indonesia: a systematic review

**DOI:** 10.1186/s12884-024-06687-6

**Published:** 2024-07-30

**Authors:** M. Syairaji, Detty Siti Nurdiati, Bayu Satria Wiratama, Zita D. Prüst, Kitty W. M. Bloemenkamp, Kim J. C. Verschueren

**Affiliations:** 1 Department of Health Information and Services, Vocational College, Universitas Gadjah Mada, Yogyakarta, Indonesia; 2 Department of Obstetrics, Birth Centre Wilhelmina’s Children Hospital, Division Women and Baby, University Medical Centre Utrecht, Utrecht, The Netherlands; 3 Department of Obstetrics and Gynecology, Faculty of Medicine, Public Health, and Nursing, Universitas Gadjah Mada, Yogyakarta, Indonesia; 4 Department of Biostatistics, Epidemiology, and Population Health, Faculty of Medicine, Public Health, and Nursing, Universitas Gadjah Mada, Yogyakarta, Indonesia; 5 Department of Obstetrics and Gynecology, Leiden University Medical Centre, Leiden, The Netherlands

**Keywords:** Maternal mortality, Systematic review, Indonesia

## Abstract

**Background:**

The maternal mortality ratio (MMR) in Indonesia is among the highest in Southeast Asia. We aim to describe trends in the MMR and causes of maternal deaths in Indonesia over the past decades, regionally and nationally.

**Methods:**

We performed a systematic review and conducted a search using PubMed, Embase, Global Health, CINAHL, Cochrane, Portal Garuda, and Google Scholar from the inception of the database to April 2023. We included all studies on the incidence and/or the causes of maternal deaths in Indonesia. The MMR was defined as the number of maternal deaths per 100,000 live births. Maternal death causes were assessed and reclassified according to the WHO International Classification of Disease Maternal Mortality (ICD-MM).

**Results:**

We included 63 studies that reported the MMR (54 studies) and/or the causes of maternal deaths (44 studies) in Indonesia from 1970 to 2022, with a total of 254,796 maternal deaths. The national MMR declined from 450 to 249 (45%) between 1990 and 2020. Great differences in MMR exist across the country, with the lowest in Java-Bali and the highest (more than twice the national MMR) in Sulawesi and Eastern Indonesia. Between 1990 and 2022, the proportion of deaths due to hemorrhage and sepsis decreased, respectively from 48 to 18% and 15–5%, while the share of deaths due to hypertensive disorders and non-obstetric causes increased, respectively from 8 to 19% and 10–49%.

**Conclusion:**

Despite the steady decline of maternal deaths in Indonesia, it remains one of the highest in Southeast Asia, with enormous disparities within the country. Hypertensive disorders and non-communicable diseases make up a growing share of maternal deaths, making maternal death reduction strategies increasingly challenging. National Maternal Death Surveillance and Response needs to be prioritized to eliminate preventable maternal deaths in Indonesia.

**Registration of systematic reviews:**

PROSPERO, CRD42022320213.

**Supplementary Information:**

The online version contains supplementary material available at 10.1186/s12884-024-06687-6.

## Background

Maternal mortality is an essential indicator of the quality of health care. The Sustainable Development Goals (SDGs) call for a global maternal mortality ratio (MMR) of less than 70 maternal deaths per 100,000 live births (LB) and an MMR of less than 140 per 100,000 live births in every country by 2030 [1]. The MMR in South-East Asia was estimated at 134 women per 100,000 live births in 2020. Indonesia has the fourth highest MMR (173/100,000 LB) in the region, following Timor-Leste, Cambodia, and Myanmar [2]. To achieve the SDGs target, the WHO recommends each country implement Maternal Death Surveillance and Response (MDSR) [3]. MDSR is a continuous cycle of identification and notification of maternal death, review of maternal death by a local maternal death review committee, analysis and interpretation of the findings, and response and monitoring of response [3].

In 1994, Indonesia implemented a policy of maternal death notification and maternal death reviews [4]. Subsequently, several programs have been implemented throughout the years to reduce maternal death in Indonesia, though no studies have evaluated the effect of these interventions on maternal outcomes [4]. The Ministry of Health (MoH) in Indonesia introduced comprehensive MDSR in 2016 [4]. Although data on all maternal deaths have been collected, no reports have been written, and little is known about the implementation and effectiveness. Reliable data on national and regional trends of maternal deaths, the causes, and understanding why the women die are essential to identify the main issues and lessons learned and assist policymakers in developing effective strategies to reduce the number of maternal deaths further.

Therefore, the primary objective of this systematic review is to analyse the national and regional MMR in Indonesia and report the MMR trend in the period before 1990 up to 2022. The secondary objective is to report the underlying causes of maternal deaths in Indonesia and the trend in causes of death in the before mentioned time frame.

## Methods

This systematic review was conducted using the Cochrane Collaboration principles and the Preferred Reporting Items for Systematic Reviews and Meta-Analysis guidelines (PRISMA) (see supplementary file 1) [5]. We registered the study protocol in PROSPERO [CRD42022320213] on May 25th, 2022.

### Setting

Indonesia is an ethnically, culturally, and economically diverse country with a population of more than 270 million people, broadly dispersed over 16,000 islands. For this review, we divided Indonesia into five regions: Java and Bali, Sumatera, Sulawesi, the Eastern part of Indonesia, and Kalimantan, each with several provinces (Fig. 2) [6].

The population density differs across the regions, as well as the density of health facilities and services, with the most health facilities in Java and Bali and the least in Sulawesi and the Eastern part of Indonesia [7, 8]. Per 100,000 people, there are 2–7 primary health centers (hereafter referred to as Puskesmas), one hospital, 50–60 doctors, two gynecologists, and 70–160 midwives. The number of health services per number of inhabitants in Java and Bali is similar to that of other regions in the country. Yet, the density of health facilities per square kilometer (km), and thus the distance and time people must travel to a health center, is significantly different [9]. The average distance to a primary health center is 3 km, and to a hospital is 12 km (0.5 km in Java and Bali, 29 km in Sulawesi) [10].

Indonesia has national health coverage, currently covering 90% of the total population [11]. The remaining 10% have private health insurance or no insurance. People with a high-middle income pay a monthly fee, while poor people are fully subsidized. Insurance covers almost all health services, including antenatal care and delivery [12].

Health care in Indonesia is based on a primary health care concept, where Puskesmas are the basic health care facilities, supported by hospitals and other community-based health facilities, from village to national level. Puskesmas offer antenatal care (ANC) and assistance with uncomplicated births and refer patients with complications to the hospital. All maternal deaths in the community and in health facilities are registered at the district health office and sent to the provincial office, and finally to the national office [13]. No data is available on the proportion of births and maternal deaths in the Puskesmas specifically.

### Search criteria

We conducted electronic searches in Pubmed, Embase, Global Health, CINAHL, Cochrane library, and Google Scholar. The search strategy consisted of keywords and MESH terms of “maternal mortality” and “Indonesia”. To ensure the inclusion of local studies, an additional search was conducted in Bahasa Indonesia in Google Scholar and Portal Garuda (supplementary file 2).

### Eligibility criteria & study selection

Studies were included if they reported the number of maternal deaths per number of live births (MMR) at the national or regional level in Indonesia and/or if they reported the causes of maternal deaths. We included all eligible studies published before the day of the electronic search on April 29th, 2023. The type of studies included in this review are quantitative observational studies, including cross-sectional (descriptive study), case-control, case-series, prospective study, retrospective study, and mixed method.

The exclusion criteria were: (1) secondary research (systematic review, commentaries, books, regulatory or committee guidance); (2) the full text unavailable; or (3) case reports or case series with less than ten maternal deaths.

**Fig. 1:**
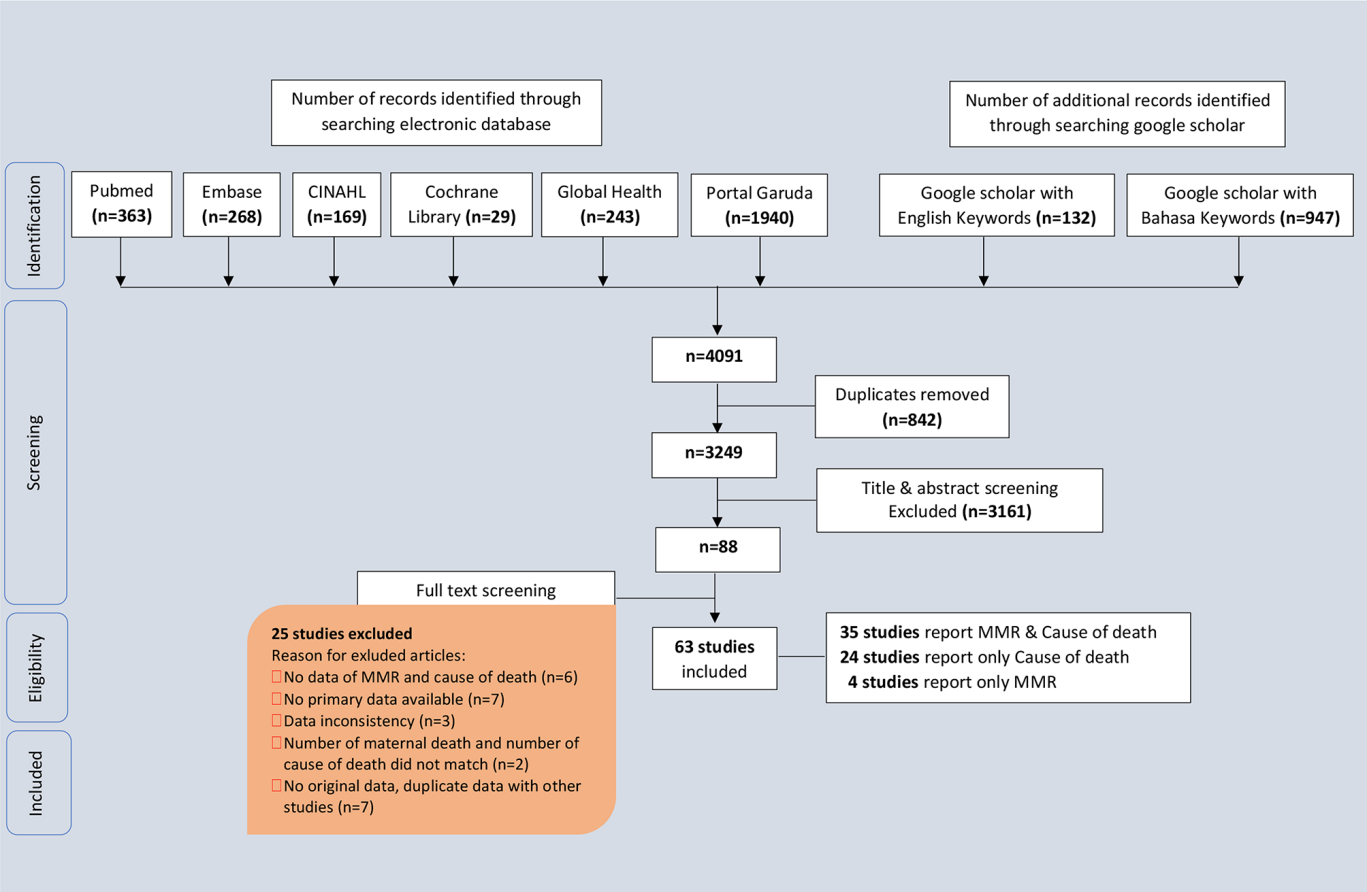
PRISMA flowchart of study selection (see supplementary file 1 for the PRISMA checklist)

We exported the yielded articles to the Mendeley reference manager and removed duplicates. Two authors (MS, BSW) screened every article independently in three steps: (1) title screening, (2) abstract screening, and (3) full-text screening. Rayyan was used for the screening process. In the case of disagreement in study selection, other authors (KV, DN) were consulted. The authors recorded the reasons for excluding studies (Fig. 1 and supplementary file 3).

**Fig. 2:**
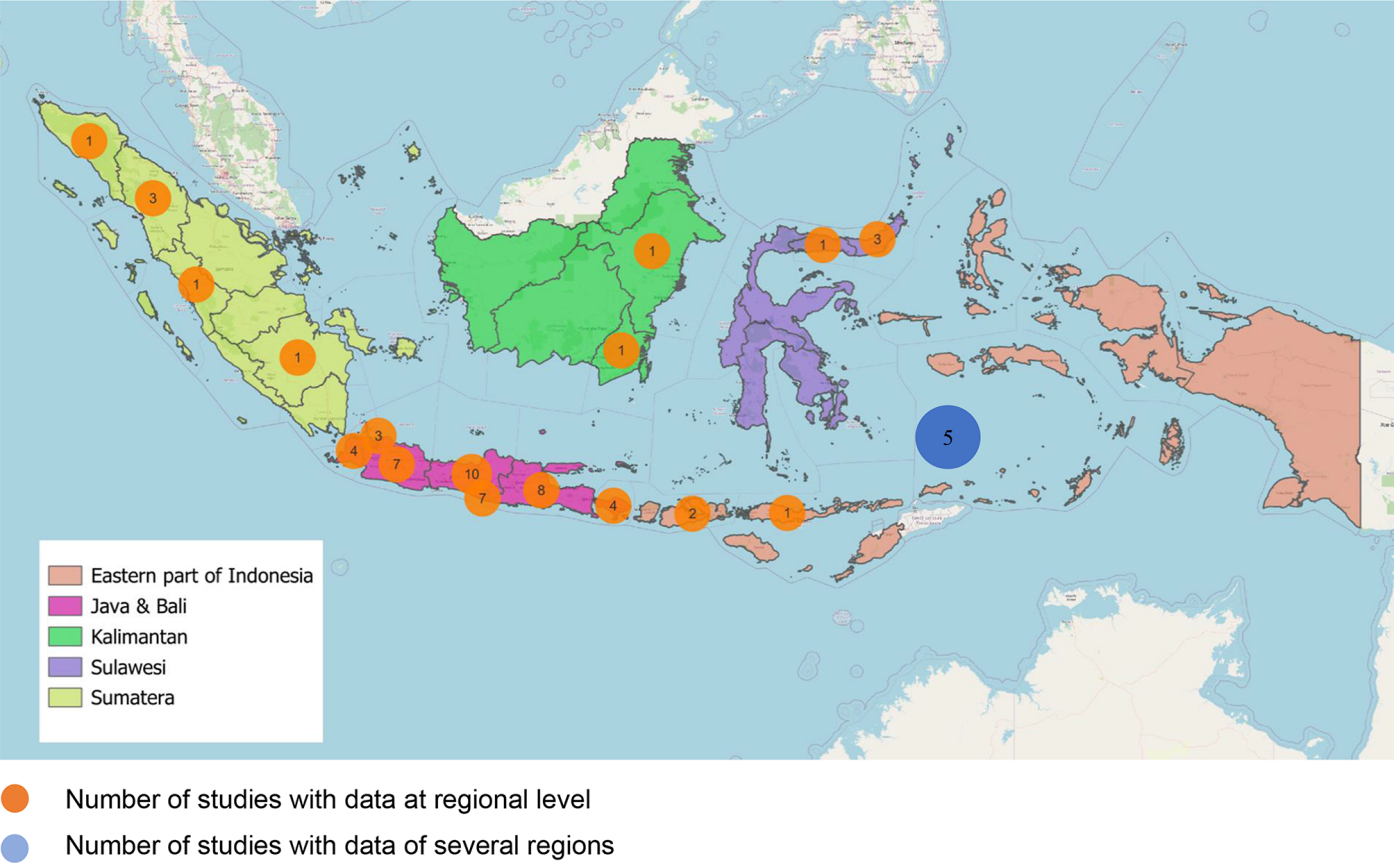
Distribution of the studies

### Data extraction

We collected data on study characteristics (title, year of publication, design study, location of study, study setting: hospital or community-based, and study period), number of maternal deaths, number of live births, MMR per 100.000 live births, and underlying causes of deaths. The included studies were divided into three groups: studies that reported the MMR and the causes of maternal deaths, studies that reported only the MMR, and studies that reported only the causes of maternal deaths.

### Quality assessment

To assess the quality of evidence, we used two assessment tools: the AXIS tool (2016) for cross-sectional studies and critical appraisal tools (JBI, 2020) for case-control studies and case series [14, 15]. Two authors (MS & BSW) independently assessed the methodological quality and risk of bias of each article. The AXIS tool consists of 20 items, and the JBI tool consists of 10 items. Each item contains one point (0 or 1). If an item was not applicable (NA) for the study, it was not scored. The obtained score was divided by the total score possible after excluding the NA items. The final score ranged from 0 to 1, categorized into very weak (0–0.20), weak (0.21–0.40), moderate (0.41–0.60), strong (0.61–0.80), and very strong (0.81–1) (see supplementary file 4).

### Definitions and summary measures

A maternal death is a woman who dies during pregnancy or within 42 days of the termination of pregnancy, irrespective of the duration and site of pregnancy, and includes both direct and indirect causes of death [16]. Coincidental deaths (such as an accident) were not considered maternal deaths. The maternal mortality ratio is the number of maternal deaths per 100,000 live births. Direct maternal deaths result from obstetric complications of pregnancy or its management, while indirect maternal deaths are those resulting from pre-existent diseases or conditions aggravated by the physiological effects of pregnancy. The causes of maternal deaths were reported according to the WHO, ICD-MM [16]. The studies reporting maternal deaths according to other classification criteria were reclassified according to the ICD-MM guideline.

We reported the data using three time frames: (1) before the Millennium Development Goals (MDGs) i.e., < 1990, (2) during MDGs from 1991 to 2015, and (3) during the SDGs from 2016 up until now. Studies with multi-year observation time, falling into two different time frames, were put in the group with the longest observation time.

### Data analysis

Descriptive statistics (percentages) were used to present the trend of maternal deaths and the causes of maternal deaths. We calculated the MMR by summing up the number of maternal deaths divided by the total live births for each time frame at a national and regional level. We used Microsoft Office Excel (Microsoft 365, version 16.70) to synthesize the data in tables, graphs, and text boxes.

## Results

We retrieved 3249 studies with our search and selected 63 studies for final analysis. Figure 1 illustrates the selection of studies throughout the different phases of this review. We found 35 studies that reported the MMR and the causes of death, four studies on only the MMR, and 24 studies on only the causes of death in Indonesia. The 63 studies included a total of 254,796 maternal deaths (from 1970 until 2022), with information on the MMR in 244,377 (96%) cases and information on the causes of deaths in 12,893 (5%) cases. Fifty-eight studies were conducted at the regional level (*n* = 43 in Java & Bali, *n* = 6 in Sumatera, *n* = 4 in Sulawesi, *n* = 3 in the Eastern part of Indonesia, and *n* = 2 in Kalimantan), and five studies at the national level. The distribution of the studies is shown in Fig. 2.

Table 1 provides an overview of all the characteristics of the included study, the reported number of maternal deaths, the MMR, and the quality assessment. A more elaborate description of the included studies and their methodological assessment can be found in supplementary files 4 and 5, respectively. Thirty-nine studies (62%) were community-based (*n* = 253,069 maternal deaths, *n* = 11,219 the causes of maternal deaths), and 24 studies (38%) were hospital-based (*n* = 1727 maternal deaths, *n* = 1674 the causes of maternal deaths). The eleven studies (18%) with a very weak or weak-rated methodological quality all had a small number of participants and comprised 0.7% of the total study population (supplementary file 4).

**Table 1:** Study characteristics reported maternal deaths and MMR, and quality assessment

No.	Authors, Year	Study design	Study setting	Province (region)	Study period	Number of maternal death	Live birth	MMR per 100.000 live birth	Quality assessment
**The MMR and the causes of deaths**									
1.	Mardjikoen, 1973 [43]	Descriptive retrospective	Hospital based (one university hospital)	Special Region of Yogyakarta (Java & Bali)	1955–1969	199	24,814	802	Moderate^∗^
2.	Fortney et al., 1988 [44]	Descriptive retrospective	Community based (one province)	Bali (Java & Bali)	1980–1982	295	38,727	762	Strong^∗^
3.	Budiarso, 1989 [45]	Prospective study	Community based (one district)	West Java (Java & Bali)	1982–1983	11	2350	468	Moderate^∗^
4.	Simbolon, 1994 [46]	Case control	Community based	DKI Jakarta (Java & Bali)	1991–1992	111	104,509	106	Strong^ς^
5.	Karkata et al., 2006 [47]	Descriptive quantitative	Hospital based (one tertiary referral hospital)	Bali (Java & Bali)	1996–2000	48	28,872	166	Moderate^∗^
6.	Ronsmans et al., 2009 [48]	Cross sectional	Hospital based (four hospitals)	Banten (Java & Bali)	2005–2006	35	36,658	95	Very strong^∗^
7.	Ocviyanti, et al., 2020 [49]	Descriptive retrospective	Hospital based (one tertiary referral hospital)	DKI Jakarta (Java & Bali)	2008–2016	159	24,054	661	Very strong^∗^
8.	Megawati, 2011 [50]	Cross sectional	Community based (one district)	West Java (Java & Bali)	2009	51	38,346	133	Strong^∗^
9.	Sunaryo et al., 2014 [51]	Cross sectional	Hospital based (one district referral hospital)	Central Java (Java & Bali)	2009–2013	128	17,610	726	Moderate^∗^
10.	Kurniati et al., 2015 [52]	Cross sectional	Community based (one district)	Special Region of Yogyakarta (Java & Bali)	2009–2013	30	29,007	103	Moderate^∗^
11.	Tejayanti et al., 2018 [53]	Descriptive quantitative	Community based (one district)	East Java (Java & Bali)	2010	49	37,738	130	Strong^∗^
12.	Aeni, 2013 [54]	Case control	Community based (one district)	Central Java (Java & Bali)	2011	24	19,048	126	Moderate^ς^
13.	Taufiqy et al., 2016 [55]	Descriptive quantitative	Hospital based (one district referral hospital)	Central Java (Java & Bali)	2011–2015	26	8645	301	Weak^∗^
14.	Prihesti et al., 2019 [56]	Case control	Hospital based (one tertiary referral hospital)	Special Region of Yogyakarta (Java & Bali)	2012–2017	100	6562	1524	Moderate^ς^
15.	Apip et al., 2019 [57]	Case control	Community based (one district)	West Java (Java & Bali)	2013–2014	67	70,355	95	Very weak^ς^
16.	Jasmiati et al., 2019 [58]	Sequential Explanatory Mixed Method	Community based (one district)	West Java (Java & Bali)	2015	68	54,828	124	Weak^∗^
17.	Prasetyo et al., 2018 [59]	Case control	Community based (eight districts)	East java (Java & Bali)	2015	125	111,968	112	Very weak^ς^
18.	Rochmatin, 2018 [60]	Non reactive research	Community based (one district)	East java (Java & Bali)	2015–2017	109	129,488	84	Strong^∗^
19.	Puspitasari et al., 2021 [61]	Descriptive quantitative	Community based (one district)	Central Java (Java & Bali)	2016–2018	77	63,896	121	Strong^∗^
20.	Indarti et al., 2021 [62]	Descriptive retrospective	Hospital based (one tertiary referral hospital)	DKI Jakarta (Java & Bali)	2016–2018	22	4226	521	Very strong^∗^
21.	Hanafi, 2019 [63]	Descriptive analytical: case series	Community based (one district)	Special Region of Yogyakarta (Java & Bali)	2016–2018	21	42,428	50	Very strong^Ω^
22.	Paramita, 2019 [64]	Case series	Community based (one district)	Special Region of Yogyakarta (Java & Bali)	2017–2018	23	25,283	91	Very strong^Ω^
23.	Atnaryan, 2021 [65]	Mixed methods: explanatory design	Community based (one district)	Special Region of Yogyakarta (Java & Bali)	2018–2019	14	15,239	92	Very strong^Ω^
24.	Pramatirta et al., 2020 [66]	Cross sectional	Hospital based (one tertiary XXXreferral hospital)	West Java (Java & Bali)	2019	36	3140	1147	Weak^∗^
25.	Muthahari, 2017 [67]	Cross sectional	Hospital based (one tertiary referral hospital)	North Sumatera (Sumatera)	2012–2016	99	2853	3470	Strong^∗^
26.	Kesty et al., 2019 [68]	Descriptive quantitative	Hospital based (one district referral hospital)	South Sumatera (Sumatera)	2013–2016	16	2191	730	Moderate^∗^
27.	Siska, 2019 [69]	Case control	Community based (one district)	West Sumatera (Sumatera)	2014–2017	32	24,616	130	Strong^ς^
28.	Tendean et al., 2021 [70]	Descriptive retrospective	Hospital based (one district referral hospital)	Gorontalo (Sulawesi)	2011–2016	76	14,068	540	Weak^∗^
29.	Lumbanraja et al., 2016 [71]	Descriptive retrospective	Hospital based (one tertiary referral hospital)	North Sulawesi (Sulawesi)	2013–2015	41	8673	473	Strong^∗^
30.	Tendean et al., 2022 [17]	Descriptive retrospective	Hospital based (one tertiary referral hospital)	North Sulawesi (Sulawesi)	2019	22	1215	1811	Moderate^∗^
31.	Tjitra et al., 1991 [72]	Prospective study	Community based (one province)	West nusa tenggara (Eastern part of Indonesia)	1986	13	966	1346	Strong^∗^
32.	Palufi et al., 2017 [73]	Descriptive quantitative	Community based (one district)	East Nusa Tenggara (Eastern part of Indonesia)	2013–2016	31	9222	336	Moderate^∗^
33.	Supratikto et al., 2002 [74]	Descriptive retrospective	Community based (three districts)	South Kalimantan (Kalimantan)	1995–1999	130	118,960	109	Moderate^∗^
34.	Mahmood et al., 2018 [75]	Descriptive retrospective	Community based (one district)	East Kalimantan (Kalimantan)	2014–2015	30	14,952	201	Strong^∗^
35.	Chi et al., 1981 [76]	Prospective study	Hospital based (12 hospitals)	Combined regions	1977–1980	135	33,701	401	Strong^∗^
**Only MMR**									
36.	Ronsmans et al., 2009 [77]	Case control	Community based (two district)	West Java (Java & Bali)	2004–2006	458	105,876	433	Moderate^ς^
37.	Josephine et al., 2014 [78]	Descriptive quantitave	Hospital based (one tertiary referral hospital)	East Java (Java & Bali)	2012	53	2849	1860	Strong^∗^
38.	Utomo et al., 2021 [79]	Surveys	Community based	Combined regions	1970	25,135	4,617,379	544	Very strong^∗^
					1975	24,776	4,966,205	499	
					1980	23,191	5,075,444	457	
					1985	21,034	5,026,855	418	
					1990	18,767	4,894,523	383	
					1995	16,698	4,744,862	352	
					2000	15,017	4,636,614	324	
					2005	13,832	4,620,847	299	
					2010	13,193	4,740,958	278	
					2015	13,120	5,032,670	261	
					2017	13,251	5,203,944	255	
39.	Kassebaum et al., 2017 [32]	Quantitave study	Community based	Combined regions	1990	18,715	4,685,779	399	Very strong^∗^
					2000	15,726	4,554,301	345	
					2015	8937	5,046,302	177	
**Only the causes of deaths**									
40.	Adisasmita et al., 2008 [80]	Cross sectional	Hospital based	Banten (Java & Bali)	2003–2004	63	–	–	Very strong^∗^
41.	Fibriana et al., 2010 [81]	Case control	Community based (one district)	Central Java (Java & Bali)	2007	30	–	–	Moderate^ς^
42.	Chomariyah, 2013 [82]	Mixed method	Hospital based (five referral hospital)	West Java (Java & Bali)	2008–2012	98	–	–	Moderate^ς^
43.	Fajarsari et al., 2012 [83]	Cross sectional	Community based (one district)	Central Java (Java & Bali)	2010–2011	103	–	–	Moderate^∗^
44.	La Banto, 2016 [84]	Descriptive quantitave	Hospital based (one general hospital)	Special Region of Yogyakarta (Java & Bali)	2013–2015	35	–	–	Weak^∗^
45.	Sulistyono et al., 2020 [85]	Retrospective study	Hospital based (one tertiary referral hospital)	East Java (Java & Bali)	2013–2015	101	–	–	Moderate^∗^
46.	Nataria et al., 2020 [86]	Sequential explanatory mixed method	Community based (one district)	Central Java (Java & Bali)	2015	48	–	–	Moderate^∗^
47.	Masturoh et al., 2017 [87]	Case control	Community based (one district)	Central Java (Java & Bali)	2016	54	–	–	Moderate^ς^
48.	Darmapatni et al., 2021 [88]	Mixed method	Community based (one province)	Bali (Java & Bali)	2016–2017	26	–	–	Moderate^∗^
49.	Astuti et al., 2021 [89]	Cross sectional	Community based (one district)	Bali (Java & Bali)	2016–2020	47	–	–	Very strong^∗^
50.	Suyanti et al., 2019 [90]	Case series	Community based (one district)	Banten (Java & Bali)	2017	58	–	–	Very strong^Ω^
51.	Desetyaputra, 2021 [91]	Retrospective study	Hospital based (one tertiary referral hospital)	East Java (Java & Bali)	2019	87	–	–	Moderate^∗^
52.	Sembiring, 2013 [92]	Retrospective study	Hospital based (one tertiary referral hospital)	North sumatera (Sumatera)	2010–2012	37	–	–	Very strong^∗^
53.	Sihombing, 2014 [93]	Case control	Community based (one district)	North sumatera (Sumatera)	2012–2013	40	–	–	Strong^ς^
54.	Safitri et al., 2018 [94]	Descriptive quantitave	Community based (one province)	Aceh (Sumatera)	2015–2016	303	–	–	Moderate^∗^
55.	Mattarungan, 2014 [95, 96]	Cross sectional	Hospital based (one tertiary referral hospital)	North sulawesi (Sulawesi)	2012	21	–	–	Weak^∗^
56.	Bahtiar, 2011 [96]	Descriptive quantitave	Community based (one district)	West nusa tenggara (Eastern part of Indonesia)	2007–2009	68	–	–	Strong^∗^
57.	Afifah et al., 2016 [97]	Follow up study	Community based	Combined regions	2011	7548	–	–	Very strong^∗^
58.	Baharuddin et al., 2019 [98]	Retrospective study	Hospital based	Combined regions	2014	90	–	–	Strong^∗^
59.	Maharani & Sutrisno, 2023 [99]	Retrospective study	Community based	East Java (Java & Bali)	2021	1138	–	–	Weak^∗^
60.	Anggondowati, et al., 2022 [100]	Cross sectional	Community based	East Java (Java & Bali)	2017–2018	103	–	–	Very strong^∗^
61.	Purwatiningsih, et al., 2023 [101]	Descriptive quantitave	Community based	Central Java (Java & Bali)	2019–2021	81	–	–	Strong^∗^
62.	Rahmadhanti & Siyam, 2023 [102]	Case control	Community based	Central Java (Java & Bali)	2022	21	–	–	Weak^ς^
63	Sakinah, et al., 2023 [103]	Mixed method	Community based	Banten (Java & Bali)	2021	254	–	–	Moderate^∗^

^*^AXIS tool; ^ς^ JBI case control; ^Ω^ JBI Case series

**Fig. 3:**
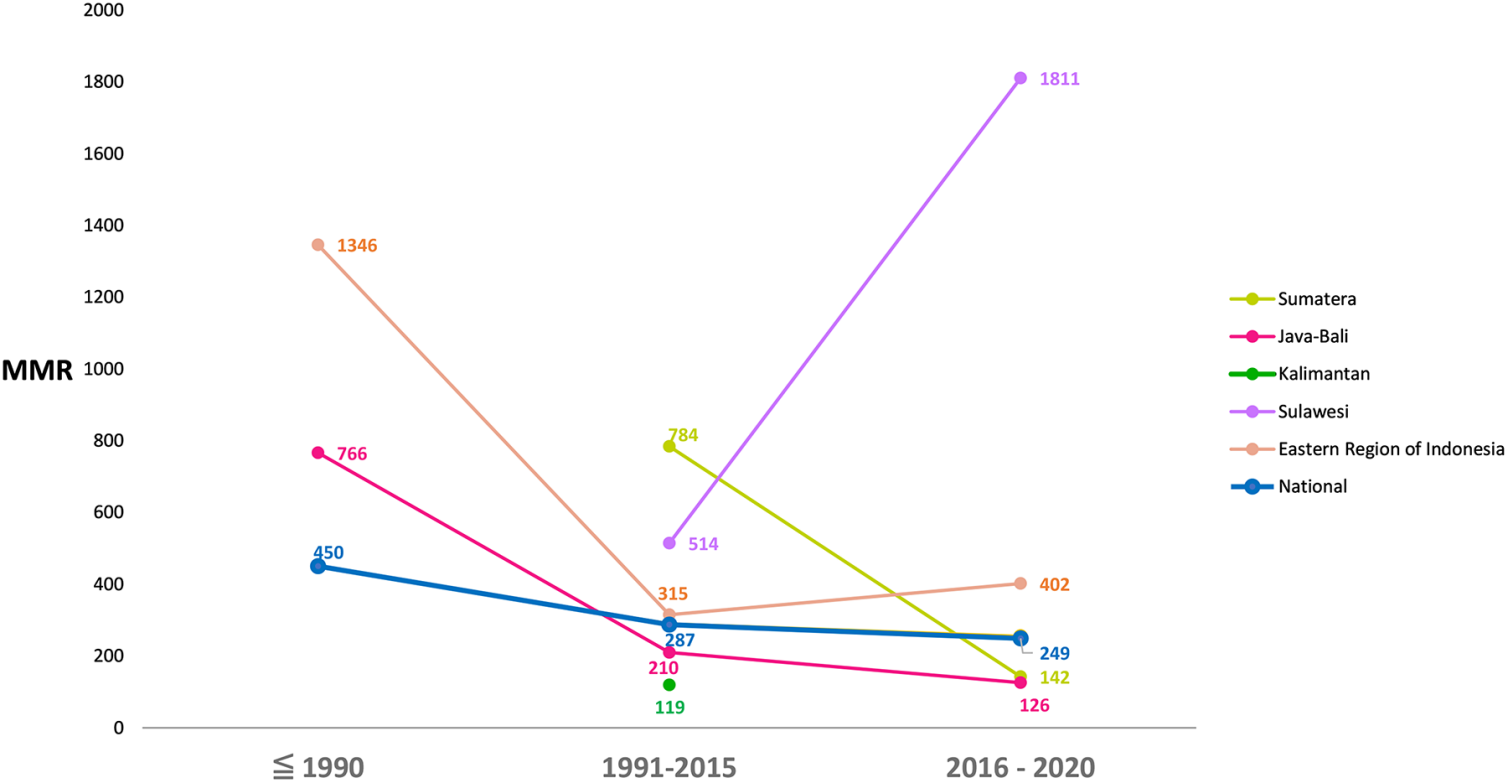
The MMR trend in Indonesia in the three-time frames, nationally and per region

Figure 3 illustrates the trends in MMR per region and time frame. The MMR in Sulawesi of 1811 per 100.000 live births is based on one study conducted in a tertiary facility and is not representative of the entire region [17]. While the MMR declined rapidly throughout all regions in Indonesia in the previous century, it has stagnated since 2015.

Table 2 demonstrates the causes of maternal deaths according to the ICD-MM. The three most common causes were non-obstetric complications (*n* = 3705, 29%), obstetric hemorrhage (*n* = 3178, 25%), and hypertensive disorders in pregnancy and childbirth (*n* = 3013, 23%). Among the 3705 cases of non-obstetric complications, 47% concerned other non-obstetric complications (*n* = 1745), 45% were non-pregnancy related infections (*n* = 1650), and 8% were cardiovascular diseases (*n* = 310) (supplementary file 6). The cause of maternal deaths was unknown/unspecified in 12% (*n* = 1525).

**Table 2:** Underlying maternal death causes in Indonesia according to the ICD-MM

	ICD-MM groups	*n* = 12,893 (100%)	Underlying cause (*n*=, %)^a^
Direct MD	1. Pregnancy with abortive outcome	236 (2%)	Abortion (*n* = 137, 1.1%) Ectopic pregnancy (*n* = 97, 0.8%) Hydatidiform mole (*n* = 2, < 0.1%)
	2. Hypertensive disorders in pregnancy, childbirth	3013 (23%)	Preeclampsia/eclampsia and HELLP syndrome (*n* = 1884, 14.6%) Gestational hypertension (*n* = 1026, 8.0%) Gestational [pregnancy-induced] oedema and proteinuria without hypertension (*n* = 103, 0.8%)
	3. Obstetric Haemorrhage	3178 (25%)	Postpartum haemorrhage (*n* = 2906, 22.5%) Antepartum haemorrhage – Unspecified antepartum haemorrhage (*n* = 154, 1.2%) – Placenta praevia (*n* = 105, 0.8%) – Placental abruption (*n* = 13, 0.1%)
	4. Pregnancy-related infection	697 (5%)	Puerperal sepsis (*n* = 352, 2.7%) Other causes of sepsis (*n* = 345, 2.7%)
	5. Other obstetric complications	516 (4%)	Unspecified (*n* = 263, 2.0%) Thrombo-embolism (*n* = 133, 1.0%) Excessive vomiting in pregnancy (*n* = 63, 0.5%) Retained placenta and membranes, w/o haemorrhage (*n* = 53, 0.4%) Suicide (*n* = 3, < 0.1%) Bladder retention (*n* = 1, < 0.1%)
	6. Unanticipated complications of management	15 (< 1%)	Anaesthesia complication (*n* = 13, 0.1%) Transfusion reaction (*n* = 2, < 0.1%)
Indirect MD	7. Non-obstetric* complications	3705 (29%)	Other maternal diseases complicating pregnancy (*n* = 1745, 13.5%) Infections, not a direct result of pregnancy (*n* = 1650, 12.8%) Pre-existing hypertension and diabetes mellitus (*n* = 310, 2.4%)
Unspecified MD	8. Unknown/undetermined	1525 (12%)	–
No MD	9. Coincidental causes	8 (< 1%)	Trauma (*n* = 8) (< 0.1%)

Legend: *n* = 59 studies, *n* = 35 studies on MMR and the cause of death, *n* = 24 studies on only the cause of death. The ICD-10 and ICD-MM guideline were used to reclassify the maternal deaths into the ICD-MM groups. More detailed overview of the indirect causes of maternal deaths of all these studies can be found in supplementary file 6

**Fig. 4:**
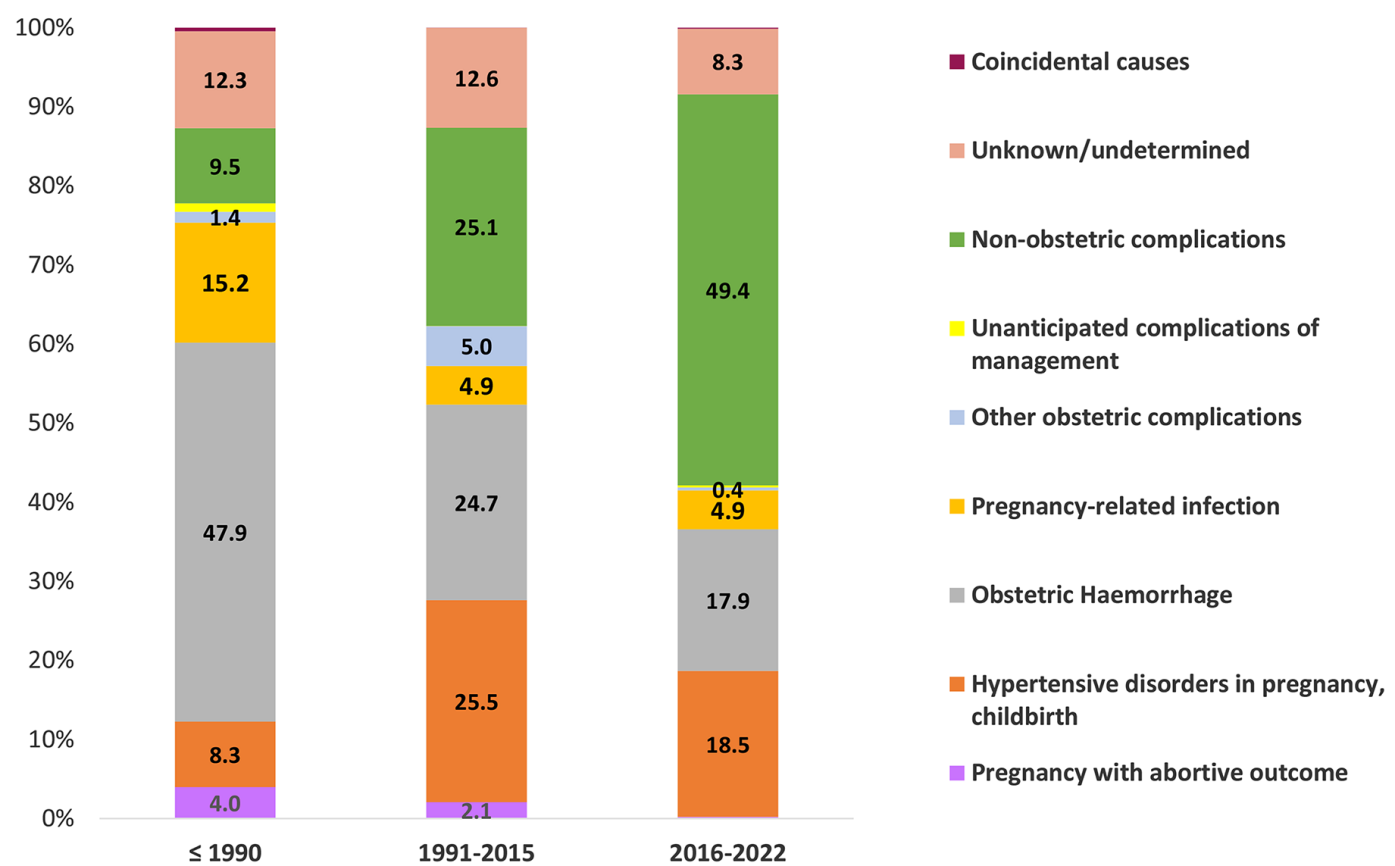
Trend of maternal death causes in Indonesia in the three-time frames, according to the ICD-MM

Figure 4 shows the trend of the causes of maternal deaths in the three timeframes. The proportion of maternal deaths due to obstetric hemorrhage and pregnancy-related infections has nearly halved in the past 30 years, respectively from 48 to 18% and from 15 to 5%. Maternal deaths due to hypertensive disorders and non-obstetric causes increased from 8 to 19% and 10–49%. A maternal death with unspecified cause was reported in 12 to 8% of cases, a proportion relatively similar throughout the years.

## Discussion

The MMR in Indonesia has declined over the past decades. The national MMR between 2016 and 2020 was 249 per 100.000 live births: a decline of 45% since 1990 and before. This systematic review illustrates a shift in the underlying causes of maternal deaths in Indonesia. While obstetric hemorrhage was the cause of almost half of the maternal deaths two decades ago, non-obstetric complications and hypertensive disorders are currently the most important causes.

The MMR in this review is higher than the estimated number from WHO (173 in 2020) and the Ministry of Health (91 in 2018–2020), most likely due to underreporting resulting from a lack of a robust maternal death surveillance system [2, 18, 19]. The underreporting is found mostly among women who died due to non-obstetric complications, often outside of a maternity ward, and misclassified as ‘no maternal deaths’ [20, 21]. The risk of an MMR estimate lower than the actual MMR is that (1) health workers and policymakers are not as aware and engaged as they can be, (2) the MMR decline (SDGs) may seem less than it actually is, and (3) if surveillance were to be improved, it will initially seem like the MMR increases, while actually, it is the reporting that is more accurate.

Most of the studies in this review were conducted in Java-Bali, a region with a lower MMR than the rest of the country. This is likely a consequence of a higher population density and a higher concentration of tertiary hospitals and research facilities. This overrepresentation may have also caused the national MMR to be underreported in our review. At the same time, Java-Bali is a referral province for complex patients, leading to a high concentration of high-risk pregnancies compared to other regions. Lack of data in some regions, such as Sulawesi and the Eastern part of Indonesia, could hinder the understanding of maternal health problems at the national level, leading to less effective policies as well as interventions in underrepresented areas.

Health facilities and medical staff are concentrated in cities. Yet, except for in Java and Bali, the most of people in Indonesia live in rural areas [8]. Access to health facilities is particularly challenging in an archipelago such as Indonesia, where the average distance to a hospital is almost 60 times more on one island than the other (Java island 0.5 km, vs. Sulawesi island 29 km), which is likely an important contributor to the disparities in pregnancy and childbirth health services and outcomes seen [10].

The high MMR in Sulawesi and the Eastern part of Indonesia can be explained by their sparse population, the lower socio-economic status of its people, the poorer infrastructure with less access to healthcare facilities, and the limited resources for medical staff (e.g. training, protocols and guidelines) [8, 22–24]. Additionally, the referral conditions vary and are generally of poor quality due to the highly decentralized health system, poor coordination and communication between primary care and referral hospitals, and lack of standardized protocols [25, 26]. A centralized national referral system can help improve uniformity, equality, and effectiveness of maternal and neonatal care among regions [25].

Implementing MDSR is especially important in Sulawesi and the Eastern part of Indonesia, as the data in these regions is the least reliable, while the MMR is the highest, and reduction strategies will be most effective. Nonetheless, multisectoral engagement, such as improving infrastructure and educational opportunities, is equally crucial to achieving maternal death reduction [27].

The MMR in Indonesia is considerably high for an upper middle-income country, highest in the Southeast Asian region, and the decline in MMR is one of the least in the region [2]. Indonesia is not likely to achieve SDG goal 3 by 2030 unless maternal death reduction strategies are prioritized, including accessible care to all, elimination of health disparities, and improved MDSR to monitor trends. The recent WHO progress report on improving maternal and newborn health indicates that globally, maternal mortality has remained stagnant or even increased in some regions. The report highlights the importance of access to high-quality and respectful maternal and newborn care to reduce maternal mortality [28]. The findings of our review align with this WHO report, as maternal mortality in Indonesia remains high despite the majority of women attending the recommended number of ANC visits and giving birth with a skilled birth attendant [29]. Strengthening the quality of care by increasing the number of facilities providing Basic Emergency Obstetric and Newborn Care and Comprehensive Emergency Obstetric and Newborn Care, especially in rural and underserved areas, in addition to referral system strengthening and expanding research capacity, is key in reducing maternal mortality in all parts of Indonesia.

The fact that the proportion of maternal deaths due to hemorrhage and sepsis decreased is in line with the increasing development of the country and the achievement of quality health care and health workers [30]. Non-obstetric complications are the most frequent causes of maternal deaths in Indonesia, similar to global maternal death proportions [31]. Similar high proportions for non-obstetric complications are reported in Sub-Sahara Africa. However, non-obstetric infections (HIV/TBC) make up the largest share there, while non-communicable diseases make up the largest in the past years [32]. Non-communicable diseases (NCD), including hypertension and obesity, are associated with increasing population density and lifestyle factors, such as low physical activity, high alcohol consumption, smoking, and an unhealthy diet. This growing impact of NCD on maternal health calls for lifestyle intervention and prevention strategies [33–35]. The recent COVID-19 pandemic has had its toll on maternal deaths in Indonesia as well, contributing to a quarter of maternal deaths due to non-obstetric complications (see supplementary file 6) due to the disease itself, and most likely even higher due to obstetric complications as the overburdened healthcare system could not provide the same level of quality care and less access to care [36, 37].

In this review, we found that pregnancy with abortive outcomes contributes to 2% (234 cases) of maternal deaths in Indonesia. However, this is likely underreported as approximately 79% of the estimated two million abortions in Indonesia are unsafe, as the practice is not legalized unless it concerns special cases (health emergencies, victims of rape) [38–40]. Providing legal, accessible, and free safe abortion services is essential to reduce maternal mortality and morbidity [39].

12% of the maternal deaths were due to unspecified causes. Attributing a cause of maternal death can be challenging, especially if multiple comorbidities are present [41]. The studies included in this review classified maternal deaths heterogeneously, complicating the comparison. Similarly, it was unclear whether the cause of death defined the underlying cause or the mode of death [42]. The implementation of maternal death audit by the local team or community, and at a national level, is crucial to improve maternal data registration, the attribution of causes, and the lessons learned to reduce maternal deaths in a ‘blame-free, shame-free’ manner.

To the best of our knowledge, this is the first and most comprehensive systematic review of trends and causes of death in Indonesia using both international and local journal databases. This study has some limitations. Firstly, the studies included in this review were very small or conducted within a certain context. Secondly, some studies might have reported the same data, considering that certain studies were conducted with a national scope within the same time frame. Thirdly, there is significant heterogeneity between the studies, for example, the use of the MMR denominator (number of live births) and causes attribution (classification system) with no specification of whether the underlying cause or mode of death is reported, limiting us in conducting a meta-analysis [42]. While reclassifying the deaths using ICD-MM enhances local and global comparability, we may have turned in the accuracy of death attribution (as we did not assess each case but only had the locally reported derivative cause).

## Conclusion

Maternal deaths in Indonesia nearly halved between 1990 and 2020, though it seems much higher than WHO estimations and considerably higher than other upper-middle income and other Southeast Asia countries. The decline of MMR is most likely a consequence of the decrease in maternal deaths due to hemorrhage and sepsis as a result of improved quality of basic obstetric care. Yet, the decline is too slow to achieve SDG 3.1 by 2030. The disparities in MMR between regions in Indonesia call for reducing health inequalities, social and economic, and travel distance. Great efforts are needed to strengthen the quality of maternal and newborn care, particularly in rural and underserved areas, improving infrastructure and accessibility, developing a national centralized referral system, and implementing incentive programs to address the unequal distribution of healthcare professionals. The share of hypertensive disorder and non-obstetric complications increased, emphasizing the importance of policymakers to steer maternal death reduction strategies more towards the prevention of non-communicable diseases and the promotion of health-related lifestyle interventions. National implementation of MDSR with a strong commitment of all stakeholders and policymakers is necessary to eliminate preventable maternal deaths and achieve the desired SDG maternal death reduction in Indonesia.

### Electronic supplementary material

Below is the link to the electronic supplementary material.

Supplementary Material 1

Supplementary Material 2

Supplementary Material 3

Supplementary Material 4

Supplementary Material 5

Supplementary Material 6

## Data Availability

Data is provided within the manuscript or supplementary information files.

## Abbreviations

MMR: Maternal Mortality Ratio
LB: Live Births
WHO: World Health Organization
MDSR: Maternal Death Surveillance and Response
MoH: Ministry of Health
PRISMA: Preferred Reporting Items for Systematic Reviews and Meta-Analysis
ANC: Antenatal Care
ICD-MM: International Classification of Disease Maternal Mortality
MDGs: Millennium Development Goals
SDGs: Sustainable Development Goals
NCD: Non-communicable diseases
