# Supplementary material for: Trends and causes of maternal mortality in Indonesia: a systematic review
Source: BMC Pregnancy Childbirth. 2024 Jul 30;24:515. doi: 10.1186/s12884-024-06687-6 (PMC11290122; doi:10.1186/s12884-024-06687-6)
Supplement: Supplementary file 1 — Supplementary Material 1 [file 12884_2024_6687_MOESM1_ESM.docx]

Supplementary file 5. Distribution of Indirect cause of death

| **Category** | **n^a^**  **(3705)** | **Sub-category (n)^a^** | **Case description (n)^a^** |
| --- | --- | --- | --- |
| Other maternal diseases classifiable elsewhere but complicating pregnancy, childbirth and the puerperium | 1745 (47%) | Other unspecified diseases and conditions (1154)(31.1%) |  |
|  |  | Diseases of the circulatory system (384)(10.4%) | Hearth disease (342)(9.2%)  Other unspecified circulatory disease (39)(1.1%)  Stroke (3)(0.1%) |
|  |  | Diseases of the respiratory system (44)(1.2%) | Pulmonary disease (20)(0.5%)  Dyspnoea/Suffocation (12)(0.3%)  Asthma (8)(0.2%)  Respiratory Failure (4)(0.1%) |
|  |  | Other specified diseases and conditions (40)(1.1%) | Congenital disorders or comorbidities (19)(0.5%)  Unspecified indirect cause (15)(0.4%)  Anaphylactic shock (3)(0.1%)  Epigastric and Symphysis Pain (2)(0.1%)  Ascites (1)(<0.1%) |
|  |  | Anaemia (31)(0.8%) |  |
|  |  | Diseases of the digestive system (23)(0.6%) | Acute fatty liver (12) (0.3%)  Liver disease (3)(0.1%)  Peritonitis (2)(0.1%)  Ileus paralytic (2)(0.1%)  Gastritis (1)(<0,1%)  Leaky Gut Syndrome(1) (<0,1%)  Intra-abdominal haemorrhage (1) (<0.1%)  Hepatic shock (1) (<0.1%) |
|  |  | Neoplasm (20)(0.5%) | Unspecified malignancy(11) (0.3%)  Breast cancer (3)(0.1%)  Brain cancer (1) (<0.1%)  Cervical cancer (1)(<0.1%)  Leukaemia (1) (<0.1%)  Rectal cancer (1) (<0.1%)  Thyroid cancer (1) (<0.1%)  Digestive neoplasm (1)(<0.1%) |
|  |  | Other diseases of the blood and blood-forming organs and certain disorders involving the immune mechanism (16)(0.4%) | Unspecified blood disorder (14)(0.4%) Idiopathic Thrombocytopenic (1) (<0.1%)  Haematological disorder (1) (<0.1%) |
|  |  | Endocrine, nutritional and metabolic diseases (15)(0.4%) | Unspecified metabolic disorder (7)(0.2 %) Hyperthyroid (4)(0.1%)  Thyroid Storm (2)(0.1%)  Refractory hypokalaemia (2)(0.1%) |
|  |  | Diseases of the musculoskeletal system and connective tissue (8)(0.2%) | Systemic Lupus Erythematosus (8) (0.2%) |
|  |  | Mental disorders and diseases of the nervous system (7)(0.2%) | Meningitis (3)(0.1%)  Neurological disorder (3) (0.1%)  Tetraplegia (1) (<0.1%) |
|  |  | Genitourinary conditions (3)(0.1%) | Chronic kidney disease (2)(0.1%)  Kidney failure (1) (<0.1%) |
| Maternal infectious and parasitic diseases classifiable elsewhere but complicating pregnancy, childbirth and the puerperium | 1650 (45%) | Other viral diseases (882)(23.8%) | Covid-19 (867)(23.4%)  Dengue Haemorrhagic Fever (DHF) (15) (0.4%) |
|  |  | Other maternal infectious and parasitic diseases (721)(19.5%) | Other unspecified infection (685)(18.5%)  Pneumonia (14)(0.4%)  Chest infections (12)(0.3%)  Intestinal infections (6)(0.2%)  Diarrhoea (3)(0.1%)  Leprosy (1) (<0.1%) |
|  |  | Human immunodeficiency [HIV] (19)(0.5%) |  |
|  |  | Tuberculosis (17)(0.5%) | Lung tuberculosis (14) (0.4%)  Meningoencephalitis Tuberculosis (2)(0.1%)  Lymphadenitis tuberculosis (1) (<0.1%) |
|  |  | Hepatitis (9)(0.2%) |  |
|  |  | Protozoal diseases (2)(0.1%) | Malaria (2)(0.1%) |
| Pre-existing hypertension | 306 (8%) |  |  |
| Diabetes mellitus in pregnancy | 4  (0.1%) |  |  |

n^a^  : Number of cases and percentage
