# Supplementary material for: Trends and causes of maternal mortality in Indonesia: a systematic review
Source: BMC Pregnancy Childbirth. 2024 Jul 30;24:515. doi: 10.1186/s12884-024-06687-6 (PMC11290122; doi:10.1186/s12884-024-06687-6)
Supplement: Supplementary file 2 — Supplementary Material 2 [file 12884_2024_6687_MOESM2_ESM.docx]

Supplementary file 4. Distribution of characteristics of study and quality assessment

|  | n (63) | Percentage (%) |
| --- | --- | --- |
| Study design |  |  |
| Case control | 11 | 17,5 |
| Case series | 3 | 4,8 |
| Census | 1 | 1,6 |
| Cross sectional | 11 | 17,5 |
| Descriptive quantitative | 13 | 20,6 |
| Descriptive retrospective | 14 | 22,2 |
| Prospective | 3 | 4,8 |
| Mixed method (case series) | 1 | 1,6 |
| Mixed method (case control) | 1 | 1,6 |
| Mixed method (cross sectional) | 4 | 6,3 |
| Surveys | 1 | 1,6 |
| Study setting |  |  |
| Community based | 39 | 61,9 |
| Hospital based | 24 | 38,1 |
| Regional/National |  |  |
| Eastern part of Indonesia | 3 | 4,8 |
| Java & Bali | 43 | 68,3 |
| Kalimantan | 2 | 3,2 |
| National and combined region | 5 | 7,9 |
| Sulawesi | 4 | 6,3 |
| Sumatera | 6 | 9,5 |
| Quality assessment |  |  |
| Very strong | 14 | 22,2 |
| Strong | 17 | 27,0 |
| Moderate | 21 | 33,3 |
| Weak | 9 | 14,3 |
| Very weak | 2 | 3,2 |
