# Supplementary material for: Trends and causes of maternal mortality in Indonesia: a systematic review
Source: BMC Pregnancy Childbirth. 2024 Jul 30;24:515. doi: 10.1186/s12884-024-06687-6 (PMC11290122; doi:10.1186/s12884-024-06687-6)
Supplement: Supplementary file 5 — Supplementary Material 5 [file 12884_2024_6687_MOESM5_ESM.docx]

**Supplementary file 1. Search strategy**

**Pubmed**

((((((((((((((((((((((((((((((((((((pregnanc*[Title/Abstract]) OR (pregnant[Title/Abstract])) OR (mother*[Title/Abstract])) OR (matern*[Title/Abstract])) OR (conception[Title/Abstract])) OR (delivery[Title/Abstract])) OR (partus[Title/Abstract])) OR (labor[Title/Abstract])) OR (labour[Title/Abstract])) OR (childbirth[Title/Abstract])) OR (caesarean[Title/Abstract])) OR (cesarean[Title/Abstract])) OR (reproductive[Title/Abstract])) OR (antenatal[Title/Abstract])) OR (prenatal[Title/Abstract])) OR (postnatal[Title/Abstract])) OR (perinatal[Title/Abstract])) OR (antepartum[Title/Abstract])) OR (peripartum[Title/Abstract])) OR (intrapartum[Title/Abstract])) OR (postpartum[Title/Abstract])) OR (post-partum[Title/Abstract])) OR (post birth[Title/Abstract])) OR (after birth[Title/Abstract])) OR (obstetric*[Title/Abstract])) OR (maternal health[MeSH Terms])) OR (maternal health services[MeSH Terms])) OR (obstetrics[MeSH Terms])) OR (maternal child nursing[MeSH Terms])) OR (pregnancy[MeSH Terms])) OR (postnatal care[MeSH Terms])) OR (prenatal care[MeSH Terms])) OR (perinatal care[MeSH Terms])) OR (antenatal care[MeSH Terms])) OR (maternal-child nursing[MeSH Terms])) OR (reproductive health[MeSH Terms]))

**AND**

(((maternal death[Title/Abstract]) OR (maternal dead[Title/Abstract])) OR (maternal mortality[Title/Abstract])) OR (maternal mortality ratio[Title/Abstract])

**AND**

((((((((((((((((((((((((((((((((((((((((((((((((((((((((((((((((((((((((((((((((((((((((indonesia) OR (aceh)) OR (banda aceh)) OR (Sumatra utara)) OR (north sumatra)) OR (medan)) OR (Sumatra barat)) OR (west sumatra)) OR (padang)) OR (riau)) OR (pekanbaru)) OR (tanjungpinang)) OR (jambi)) OR (bengkulu)) OR (Sumatra selatan)) OR (south sumatra)) OR (palembang)) OR (bangka belitung)) OR (pangkalpinang)) OR (lampung)) OR (banten)) OR (serang)) OR (jawa barat)) OR (west java)) OR (bandung)) OR (jakarta)) OR (jawa tengah)) OR (center java)) OR (semarang)) OR (yogyakarta)) OR (jawa timur)) OR (east java)) OR (bali)) OR (denpasar)) OR (nusa tenggara barat)) OR (west nusa tenggara)) OR (mataram)) OR (nusa tenggara timur)) OR (east nusa tenggara)) OR (kupang)) OR (Kalimantan barat)) OR (west kalimantan)) OR (west borneo)) OR (west kalimantan)) OR (pontianak)) OR (Kalimantan selatan)) OR (south kalimantan)) OR (banjarmasin)) OR (Kalimantan tengah)) OR (center borneo)) OR (center kalimantan)) OR (palangkaraya)) OR (Kalimantan timur)) OR (east kalimantan)) OR (east borneo)) OR (samarinda)) OR (Kalimantan utara)) OR (north kalimantan)) OR (north borneo)) OR (tanjong selor)) OR (gorontalo)) OR (Sulawesi barat)) OR (west sulawesi)) OR (west celebes)) OR (mamuju)) OR (Sulawesi selatan)) OR (south Sulawesi)) OR (south celebes)) OR (makasar)) OR (Sulawesi tengah)) OR (center sulawesi)) OR (center celebes)) OR (palu)) OR (Sulawesi tenggara)) OR (southeast sulawesi)) OR (southeast celebes)) OR (kendari)) OR (Sulawesi utara)) OR (north sulawesi)) OR (north celebes)) OR (manado)) OR (maluku)) OR (moluccas)) OR (ambon)) OR (north maluku)) OR (north moluccas)) OR (papua)) OR (west papua)) OR (jayapura)

Date of search : April 29^th^ 2023

Results : 363

**CINAHL**

AB (pregnanc* OR pregnant OR mother* OR matern* OR conception OR delivery OR partus OR labor OR labour OR childbirth OR caesarian OR cesarian OR section OR puerper* OR reproductive OR sexual health OR antenatal OR prenatal OR postnatal OR perinatal OR antepartum OR peripartum OR intrapartum OR postpartum OR post-partum OR post birth OR after birth OR obstetric* OR maternal health OR maternal health services OR obstetrics OR maternal child nursing OR pregnancy OR postnatal care OR prenatal care OR perinatal care OR antenatal care OR maternal-child nursing OR reproductive health)

**AND**

AB (maternal death OR maternal dead OR maternal mortality OR maternal mortality ratio)

**AND**

TX (Indonesia OR aceh OR banda aceh OR sumatra utara OR north sumatra OR medan OR sumatra barat OR west sumatra OR padang OR riau OR pekanbaru OR tanjungpinang OR jambi OR bengkulu OR sumatra selatan OR south sumatra OR palembang OR bangka belitung OR pangkalpinang OR lampung OR banten OR serang OR jawa barat OR west java OR bandung OR jakarta OR jawa tengah OR center java OR semarang OR yogyakarta OR jawa timur OR east java OR bali OR denpasar OR nusa tenggara barat OR west nusa tenggara OR mataram OR nusa tenggara timur OR east nusa tenggara OR kupang OR kalimantan barat OR west kalimantan OR west borneo OR west kalimantan OR pontianak OR kalimantan selatan OR south kalimantan OR Banjarmasin OR kalimantan tengah OR center borneo OR center kalimantan OR palangkaraya OR kalimantan timur OR east kalimantan OR east borneo OR samarinda OR kalimantan utara OR north kalimantan OR north borneo OR tanjung selor OR Gorontalo OR sulawesi barat OR west sulawesi OR west celebes OR mamuju OR sulawesi selatan OR south sulawesi OR south celebes OR makasar OR sulawesi tengah OR center sulawesi OR center celebes OR palu OR sulawesi tenggara OR southeast sulawesi OR southeast celebes OR Kendari OR sulawesi utara OR north sulawesi OR north celebes OR manado OR maluku OR moluccas OR ambon OR north maluku OR north moluccas OR papua OR west papua OR jayapura)

Date of search : April 29^th^ 2023

Results : 169

**Embase**

(pregnanc*:ab,ti OR pregnant:ab,ti OR mother*:ab,ti OR matern*:ab,ti OR conception:ab,ti OR delivery:ab,ti OR partus:ab,ti OR labor:ab,ti OR labour:ab,ti OR childbirth:ab,ti OR caesarian:ab,ti OR cesarian:ab,ti OR section:ab,ti OR puerper*:ab,ti OR reproductive:ab,ti OR ‘sexual health’:ab,ti OR antenatal:ab,ti OR prenatal:ab,ti OR postnatal:ab,ti OR perinatal:ab,ti OR antepartum:ab,ti OR peripartum:ab,ti OR intrapartum:ab,ti OR postpartum:ab,ti OR post-partum:ab,ti OR ‘post birth’:ab,ti OR ‘after birth’:ab,ti OR obstetric*:ab,ti OR ‘maternal health‘/exp OR ‘maternal health services’/exp OR obstetrics/exp OR ‘maternal child nursing’/exp OR pregnancy/exp OR ‘postnatal care’/exp OR ‘prenatal care’/exp OR ‘perinatal care’/exp OR ‘antenatal care’/exp OR ‘maternal-child nursing’/exp OR 'reproductive health’/exp)

**AND**

(‘maternal death’:ab,ti OR ‘maternal dead’:ab,ti OR ‘maternal mortality’:ab,ti OR ‘maternal mortality ratio’:ab,ti)

**AND**

(indonesia OR aceh OR ‘banda aceh’ OR ‘sumatra utara’ OR ‘north sumatra’ OR medan OR ‘sumatra barat’ OR ‘west sumatra’ OR padang OR riau OR pekanbaru OR tanjungpinang OR jambi OR bengkulu OR ‘sumatra selatan’ OR ‘south sumatra’ OR palembang OR ‘bangka belitung’ OR pangkalpinang OR lampung OR banten OR serang OR ‘jawa barat’ OR ‘west java’ OR bandung OR jakarta OR ‘jawa tengah’ ‘center java’ OR semarang OR yogyakarta OR ‘jawa timur’ OR ‘east java’ OR bali OR denpasar OR ‘nusa tenggara barat’ OR ‘west nusa tenggara’ OR mataram OR ‘nusa tenggara timur’ OR ‘east nusa tenggara’ OR kupang OR ‘kalimantan barat’ OR ‘west kalimantan’ OR ‘west borneo’ OR ‘west kalimantan’ OR pontianak OR ‘kalimantan selatan’ OR ‘south kalimantan’ OR banjarmasin OR ‘kalimantan tengah’ OR ‘center borneo’ OR ‘center kalimantan’ OR palangkaraya OR ‘kalimantan timur’ OR ‘east kalimantan’ OR ‘east borneo’ OR samarinda OR ‘kalimantan utara’ OR ‘north kalimantan’ OR ‘north borneo’ OR ‘tanjung selor’ OR gorontalo OR ‘sulawesi barat’ OR ‘west sulawesi’ OR ‘west celebes’ OR mamuju OR ‘sulawesi selatan’ OR ‘south sulawesi’ OR ‘south celebes’ OR makasar OR ‘sulawesi tengah’ OR ‘center sulawesi’ OR ‘center celebes’ OR palu OR ‘sulawesi tenggara’ OR ‘southeast sulawesi’ OR ‘southeast celebes’ OR kendari OR ‘sulawesi utara’ OR ‘north sulawesi’ OR ‘north celebes’ OR manado OR maluku OR moluccas OR ambon OR ‘north maluku’ OR ‘north moluccas’ OR papua OR ‘west papua’ OR jayapura)

Date of search : April 29^th^ 2023

Results : 268

**Global Health**

(ab:(pregnanc*) OR ab:(pregnant) OR ab:(mother*) OR ab:(matern*) OR ab:(conception) OR ab:(delivery) OR ab:(partus) OR ab:(labor) OR ab:(labour) OR ab:(childbirth) OR ab:(caesarian) OR ab:(cesarian) OR ab:(section) OR ab:(puerper*) OR ab:(reproductive) OR ab:(“sexual health”) OR ab:(antenatal) OR ab:(prenatal) OR ab:(postnatal) OR ab:(perinatal) OR ab:(antepartum) OR ab:(peripartum) OR ab:(intrapartum) OR ab:(postpartum) OR ab:(“post-partum”) OR ab:(“post birth”) OR ab:(“after birth”) OR ab:(obstetric*) OR ab:( “maternal health”) OR ab:(“maternal health services”) OR ab:(obstetrics) OR ab:(“maternal child nursing”) OR ab:(pregnancy) OR ab:(“postnatal care”) OR ab:(“prenatal care”) OR ab:(“perinatal care”) OR ab:( “antenatal care”) OR ab:(“maternal-child nursing”) OR ab:(“reproductive health”))

**AND**

(ab:(“maternal death”) OR ab:(“maternal dead”) OR ab:(“maternal mortality”) OR ab:(“maternal mortality ratio”))

**AND**

((Indonesia) OR (aceh) OR (“banda aceh”) OR (“sumatra utara”) OR (“north sumatra”) OR (medan) OR (“sumatra barat”) OR (“west sumatra”) OR (padang) OR (riau) OR (pekanbaru) OR (tanjungpinang) OR (jambi) OR (bengkulu) OR (“sumatra selatan”) OR (“south sumatra”) OR (palembang) OR (“bangka belitung”) OR (pangkalpinang) OR (lampung) OR (banten) OR (serang) OR (“jawa barat”) OR (“west java”) OR (bandung) OR (jakarta) OR (“jawa tengah”) OR (“center java”) OR (semarang) OR (yogyakarta) OR (“jawa timur”) OR (“east java”) OR (bali) OR (denpasar) OR (“nusa tenggara barat”) OR (“west nusa tenggara”) OR (mataram) OR (“nusa tenggara timur”) OR (“east nusa tenggara”) OR (kupang) OR (“kalimantan barat”) OR (“west kalimantan”) OR (“west borneo”) OR (“west kalimantan”) OR (pontianak) OR (“kalimantan selatan”) OR (“south kalimantan”) OR (Banjarmasin) OR (“kalimantan tengah”) OR (“center borneo”) OR (“center kalimantan”) OR (palangkaraya) OR (“kalimantan timur”) OR (“east kalimantan”) OR (“east borneo”) OR (samarinda) OR (“kalimantan utara”) OR (“north kalimantan”) OR (“north borneo”) OR (“tanjung selor”) OR (Gorontalo) OR (“sulawesi barat”) OR (“west sulawesi”) OR (“west celebes”) OR (mamuju) OR (“sulawesi selatan”) OR (“south sulawesi”) OR (“south celebes”) OR (makasar) OR (“sulawesi tengah”) OR (“center sulawesi”) OR (“center celebes”) OR (palu) OR (“sulawesi tenggara”) OR (“southeast sulawesi”) OR (“southeast celebes”) OR (Kendari) OR (“sulawesi utara”) OR (“north sulawesi”) OR (“north celebes”) OR (manado) OR (maluku) OR (moluccas) OR (ambon) OR (“north maluku”) OR (“north moluccas”) OR (papua) OR (“west papua”) OR (jayapura))

Date of search : April 29^th^ 2023

Results : 243

**Cochrane library**

(pregnanc*):ti,ab,kw OR (pregnant):ti,ab,kw OR (mother*):ti,ab,kw OR (matern*):ti,ab,kw OR (conception):ti,ab,kw OR (delivery):ti,ab,kw OR (partus):ti,ab,kw OR (labor):ti,ab,kw OR (labour):ti,ab,kw OR (childbirth):ti,ab,kw OR (caesarean):ti,ab,kw OR (cesarean):ti,ab,kw OR (puerper*):ti,ab,kw OR (reproductive):ti,ab,kw OR (antenatal):ti,ab,kw OR (prenatal):ti,ab,kw OR (postnatal):ti,ab,kw OR (perinatal):ti,ab,kw OR (antepartum):ti,ab,kw OR (peripartum):ti,ab,kw OR (intrapartum):ti,ab,kw OR (postpartum):ti,ab,kw OR (“post-partum”):ti,ab,kw OR (“post birth”):ti,ab,kw OR (“after birth”):ti,ab,kw OR (obstetric*):ti,ab,kw

**AND**

(“maternal death”):ti,ab,kw OR (“maternal dead”):ti,ab,kw OR (“maternal mortality”):ti,ab,kw OR (“maternal mortality ratio”):ti,ab,kw

**AND**

Indonesia OR aceh OR “banda aceh” OR “sumatra utara” OR “north Sumatra” OR medan OR “sumatra barat” OR “west Sumatra” OR padang OR riau OR pekanbaru OR tanjungpinang OR jambi OR bengkulu OR “sumatra selatan” OR “south sumatra” OR palembang OR “bangka Belitung” OR pangkalpinang OR lampung OR banten OR serang OR “jawa barat” OR “west java” OR bandung OR jakarta OR “jawa tengah” OR “center java” OR semarang OR yogyakarta OR “jawa timur” OR “east java” OR bali OR denpasar OR “nusa tenggara barat” OR “west nusa tenggara” OR mataram OR “nusa tenggara timur” OR “east nusa tenggara” OR kupang OR “kalimantan barat” OR “west Kalimantan” OR “west borneo” OR “west Kalimantan” OR pontianak OR “kalimantan selatan” OR “south Kalimantan” OR Banjarmasin OR “kalimantan tengah” OR “center borneo” OR “center Kalimantan” OR palangkaraya OR “kalimantan timur” OR “east Kalimantan” OR “east borneo” OR samarinda OR “kalimantan utara” OR “north Kalimantan” OR “north borneo” OR “tanjung selor” OR Gorontalo OR “sulawesi barat” OR “west Sulawesi” OR “west Celebes” OR mamuju OR “sulawesi selatan” OR “south Sulawesi” OR “south Celebes” OR makasar OR “sulawesi tengah” OR “center Sulawesi” OR “center Celebes” OR palu OR “sulawesi tenggara” OR “southeast Sulawesi” OR “southeast Celebes” OR Kendari OR “sulawesi utara” OR “north Sulawesi” OR “north Celebes” OR manado OR maluku OR moluccas OR ambon OR “north maluku” OR “north moluccas” OR papua OR “west papua” OR jayapura

Date of search : April 29^th^ 2023

Results : 29

**Google scholar (English)**

(“pregnancy” OR “pregnant” OR “mother” OR “maternal” OR “delivery”) (“dead” OR “death” OR “mortality”) (“Indonesia”)

Date of search : April 29^th^ 2023

Results : 132

**Google scholar (Bahasa Indonesia)**

(“Ibu” OR “maternal” OR “hamil” OR “melahirkan” OR “persalinan” OR “nifas” OR “sectio caesarea” OR “antenatal” OR “prenatal” OR “postnatal”) (“kematian”)

Date of search : April 29^th^ 2023

Results : 947

**Portal Garuda**

Kematian Ibu

Date of search : April 29^th^ 2023

Results : 1940
