# Supplementary material for: Trends and causes of maternal mortality in Indonesia: a systematic review
Source: BMC Pregnancy Childbirth. 2024 Jul 30;24:515. doi: 10.1186/s12884-024-06687-6 (PMC11290122; doi:10.1186/s12884-024-06687-6)
Supplement: Supplementary file 6 — Supplementary Material 6 [file 12884_2024_6687_MOESM6_ESM.docx]

**Supplementary file 2. Characteristics of excluded studies from full text screening**

| No. | Authors, Year | Title | Study design | Study aims | Reason for exclusion |
| --- | --- | --- | --- | --- | --- |
| 1. | Jayanti et.al, 2017 | Faktor Yang Memengaruhi Kematian Ibu (Studi Kasus Di Kota Surabaya) | Case control | To determine risk factors of maternal mortality in Surabaya | Data intertwined with another included article |
| 2. | Andini et.al, 2016 | GAMBARAN ANGKA KEMATIAN IBU DI RSUP. Prof. Dr. RD KANDOU MANADO PERIODE JANUARI 2014â€“SEPTEMBER 2015 | Retrospective descriptive | to determine the description of Maternal Mortality Rate (MMR) in RSUP Prof. Dr. dr. R. D. Kandou Manado period January 2014 - September 2015 | Data intertwined with another included article |
| 3. | Suwant1 et.al,  2002 | Hubungan Kualitas Perawatan Kehamilan Dan Kualitas Pertolongan Persalinan Dengan Kematian Maternal Di Kabupaten Klaten | Case control | To examine the relationship between quality of the antenatal care and quality of labor service with the maternal mortality in Klaten district | No data of MMR and cause of death |
| 4. | Soto et.al, 2013 | Investment Case For Improving Maternal And Child Health: Results From Four Countries. | Problem solving workshop and cohort model for estimating costs and impact | Focus on the results of the analysis of investment case on maternal and child health as they pertain to a select number of disadvantaged districts and cities in this study countries | No data of MMR and cause of death |
| 5. | Nurdiana et.al, 2019 | Memahami Tren Penyebab Kematian Ibu Dengan Menggunakan Data Audit Maternal Di Kabupaten Karawang, Indonesia | Descriptive quantitave | To describe maternal death which obtained from maternal audit documents in Karawang Regency | Data inconsistency |
| 6. | Masturoh et.al, 2019 | PATH ANALISIS : TIGA KETERLAMBATAN PENYEBAB KEMATIAN MATERNAL DI KABUPATEN BREBES | Case control | Unclear | Same author and data with article ID 630 (duplicate with different title) |
| 7. | Ika et.al, 2007 | Faktor-Faktor Risiko Yang Mempengaruhi Kematian Maternal (Studi Kasus Di Kabupaten Cilacap) | Case control | To determine the risk factors that influence maternal mortality, which consist close determinants, intermediate determinants and far determinants. | Data inconsistency |
| 8. | Hardhantyo et.al, 2020 | Impact Of The Sister Hospital Program On Maternal Mortality And Neonatal Mortality In Nusa Tenggara Timur Province, Indonesia | Longitudinal ecological study design | To evaluate the impact of the Sister Hospital program on MMRs and NMRs in 2009–2017 | No primary data available |
| 9. | Aryanty et.al, 2021 | Contraceptive Use And Maternal Mortality In Indonesia: A Community-Level Ecological Analysis | Ecological study | To examine the correlation between contraceptive use and maternal mortality in Indonesia | No data of MMR and cause of death |
| 10. | D'Ambruoso et.al, 2010 | A Lost Cause? Extending Verbal Autopsy To Investigate Biomedical And Socio-Cultural Causes Of Maternal Death In Burkina Faso And Indonesia. | Surveys | To extend the standard VA (interview and analytical) schedules to gather and present additional information on experiences of emergency care-seeking, from the perspectives of family members who experienced some or all of the relevant events; to identify biomedical and socio-cultural factors relevant to outcomes, and; to present an illustrative analysis that considers the utility of the extension for routine health planning and surveillance. | No data of MMR and cause of death |
| 11. | Siswosudarmo,  2014 | Effect Of Delay In Postpartum Hemorrhage Management On The Rate Of Near-Miss And Maternal Death Cases: Pengaruh Keterlambatan Penanganan Perdarahan Pascapersalinan Terhadap Kejadian Hampir Mati Dan Kematian Ibu | Prospective cohort | To recognize the effect of delay in the management of postpartum bleeding to the occurrence of near-miss and maternal death cases. | No data of MMR and cause of death |
| 12. | Pujana, 2008 | Determinan Kematian Ibu Provinsi Bali Tahun 2006-2007 | Case control | To determine the determinants of maternal mortality in Bali Province in 2006-2007 | No data of MMR and cause of death |
| 13. | Tejayanti et.al, 2012 | Disparitas akses dan kualitas: Kajian determinan kematian maternal di lima region Indonesia | Secondary data study | To determine maternal cause of death based on follow up study of national census 2010 | No primary data available |
| 14. | Sandi, 2018 | PREEKLAMSIA DAN KEMATIAN MATERNAL DI RSUP DR. SARDJITO | Cross sectional | To investigate the relationship between risk factors of maternal mortality with preeclampsia in Dr Sardjito Hospital from 2012 until September 2017 | Data intertwined with another included article |
| 15. | Budiarso et.al, 1991 | Estimasi Kematian Maternal Dengan Metoda Saudara Kandung Perempuan Di Jawa Barat (Sisterhood Method) | Retrospective study | To estimate maternal mortality using the sisterhood Method in West Java | Data inconsistency |
| 16. | Brahmantya, 2018 | HUBUNGAN ANTARA METODE PERSALINAN DENGAN KEMATIAN IBU DI RSUP DR. SARDJITO YOGYAKARTA | Cross sectional | To know the relationship between delivery method and maternal mortality in RSUP dr. Sardjito Yogyakarta | Data intertwined with another included article |
| No | **Authors, Year** | **Title** | **Study design** | **Study aims** | **Reason for exclusion** |
| 17. | Fibriana, 2007 | Faktor-Faktor Risiko Yang Mempengaruhi Kematian Maternal | Case control | To know the risk factors that influence maternal mortality, which consist of proximate determinant, intermediate determinant and distant determinant. | Not all maternal death case included, number of maternal death and number of cause of death not match |
| 18. | Budiarso et.al, 2019 | Kematian Maternal Dan Pelayanan Kesehatan, Survei Demografi Dan Kesehatan Indonesia, 1994 | Surveys | To describe maternal mortality and health services in 1994 | No primary data available |
| 19. | Fibriana, 2007 | Faktor-Faktor Risiko Yang Mempengaruhi Kematian Maternal (Studi Kasus Di Kabupaten Cilacap)(Arulita Ika Fibriana Risk Factors That Influence Maternal Mortality (Case Study At Cilacap District) | Case control | To know the risk factors that influence maternal mortality, which consist of proximate determinant, intermediate determinant and distant determinant. | Not all maternal death case included, number of maternal death and number of cause of death not match |
| 20. | Kwast, 1996 | Reduction Of Maternal And Perinatal Mortality In Rural And Peri-Urban Settings: What Works? | Surveys | To lay out conceptual frameworks for programming in the fields of maternal and neonatal health for the reduction of maternal and peri/neonatal mortality; to describe selected MotherCare demonstration projects in the first 5 years between 1989 and 1993 in Bolivia, Guatemala, Indonesia and Nigeria | No primary data available |
| 21. | Scott et.al, 2013 | Maternal Mortality, Birth With A Health Professional And Distance To Obstetric Care In Indonesia And Bangladesh | Retrospective cohort | To examine the relationship between distance to a health facility, consulting a health professional and maternal mortality | No primary data available |
| 22. | Saifuddin, 2006 | Kematian Ibu Di Indonesia Dapatkah Kita Mencapai Target Mdgs 2015? | No information | No information | No primary data available |
| 23. | Jayanti, 2016 | INDEKS PREDIKTIF RISIKO KEMATIAN IBU DI KOTA SURABAYA | Case control | To determine the risk factors predictive index of maternal mortality in Surabaya city | Same author and data with article ID 32 (duplicate with different title) |
| 24. | Cameron et.al, 2019 | Understanding the determinants of maternal mortality: An observational study using the Indonesian Population Census | Observational analytic | To test the hypothesis that health service access and quality are important determinants of maternal death and explain the differences between high maternal mortality and low maternal mortality provinces | Data intertwined with another included article |
| 25. | Awang et.al, 2022 | Study Of Maternal Death In Urban And Rural Based On Pregnancy Planning In East Sumba Regency 2014-2018 | Observational analytic | Assessing Maternal Mortality and the Role of Family Planning (KB) in order to reduce MMR in East Sumba Regency in 2014 – 2018. Specifically: To identify the relationship between maternal mortality and Pregnancy Planning in East Sumba Regency in 2014 – 2018 | No primary data available |
